# Structure evaluation of the implementation of geriatric models in primary care: a multiple-case study of models involving advanced geriatric nurses in five municipalities in Norway

**DOI:** 10.1186/s12913-020-05566-y

**Published:** 2020-08-14

**Authors:** Konstantinos Antypas, Marit Kirkevold

**Affiliations:** 1 Department of Nursing Science, Institute of Health and Society, Faculty of Medicine, University of Oslo, P.O Box 124, 0314 Oslo, Blindern Norway; 2 SINTEF Digital, Oslo, Norway; 3 Head of Institute of Nursing and Health Promotion, Faculty of Health Sciences, Oslo Metropolitan University, Oslo, Norway

**Keywords:** Advanced nursing practice, Advanced geriatric nursing, Model of care, Primary healthcare, Care of older adults

## Abstract

**Background:**

The Advanced Geriatric Nurse role recently has been introduced into Norway’s primary healthcare system, and our study’s purpose was to examine the implementation of models of care developed for Advanced Geriatric Nurse in primary care. With a structure evaluation, we tried to identify conditions that affect the implementation of different models of care and understand how these conditions affected the realisation of each model’s intentions and goals.

**Methods:**

An embedded multiple-case study was used that included five Norwegian municipalities and seven AGNs. The study included data from August 2014 through September 2018. We used data from 25 semi-structured face-to-face interviews with AGNs and stakeholders, documents and statistical information. We used a cross-case procedure with an emphasis on case findings for the analysis of the multiple case study.

**Results:**

We analysed the structure-related conditions on two levels: the meso-level and the micro-level. On the meso-level, we found that the conditions that affected the implementation of the different models of care were related to each municipality’s structure characteristics, stakeholders’ involvement in the design of the models of care, the clarity of the models and their goals, the evaluation of the models and their adaptation. At the micro-level, we found that the conditions that affected the models’ implementation were related to the collaboration within the implemented models of care, the role clarity of Advanced Geriatric Nurses themselves and adjustments within the models.

**Conclusions:**

The implementation of the AGN role in Norway seems to have been implemented in ways that can impact patients and municipalities positively. Potential improvements include extensive stakeholder involvement, improved roles, goal clarity and better documentation of structures and outcomes. The models’ dynamic nature seemed to be a beneficial characteristic, but adaptation should be systematic and a necessary time should be considered for a new model of care to be integrated and produce results.

## Introduction

Primary healthcare in many countries is facing several challenges as the population ages and those who live longer acquire one or more diseases [1–3]. As a response to these challenges, new models of care have emerged, often including Advanced Nursing Practice (ANP) roles [1]. ANPs usually are master’s-prepared registered nurses with expert knowledge and skills for complex decision making and clinical competencies for expanded practice [4]. ANPs in primary care can manage up to 80% of the patients’ needs independently [5] while providing high-quality care [6–8]. In elderly care, ANPs can reduce readmissions and increase satisfaction [9], and in long-term geriatric care, they lowered rates of depression, urinary incontinence, pressure ulcers, restraint use and aggressive behaviours [10]. The implementation of the ANP roles is closely related to the model of care through which they will be introduced [11, 12]. Model of care is the overarching design that provide a particular type of healthcare service and is defined by the stakeholder roles and relationships involved in the model [11–13]. Many studies have been published on the implementation of ANP roles [1, 4, 9, 11, 12, 14–17], and some on the evaluation of the models of care [18–20]. Our study intends to document and evaluate how the models of care that include ANPs in primary care for older people have been implemented, since this is essential for the future development of the models of care and for informing their successful large-scale implementation.

In Norway, the setting of our study, the Advanced Nurse Practitioner (ANP) role –– and more specifically, the Advanced Geriatric Nurse (AGN) role –– was introduced in primary healthcare in 2011. Similarly to many other countries [1, 21], the challenges of the primary healthcare in Norway include limited financial capacity [22], scarcity of competence [23] and limited resources by the General Practitioners (GP) [24, 25], and the AGN role can have a significant contribution in facing these challenges. Research on the implementation of the models of care that include AGNs in Norway can be relevant for other countries that are introducing their own ANP roles through respective models of care. Furthermore, the knowledge produced can provide new perspectives for models of care and ANP roles that already are established. Those environments might be more hesitant to experiment and innovate compared with the Norwegian municipalities that had to implement these models of care and the new role from the beginning. Finally, this study can contribute to existing theory by providing insights into the practical implementation of the models of care that include ANP roles [11, 12].

### Background

The framework for the Participatory Evidence-based Patient-centred Process for ANP role development, implementation and evaluation (PEPPA) has been used for implementing new roles and planning or interpreting research, as well as in practice settings and policy contexts [11, 26]. PEPPA-Plus—a later version of PEPPA—included guidance for evaluating ANP roles and manifested the complexity of the task by discretising the stages of ANP role development and the assessment dimensions [12]. Both frameworks emphasise the importance of the models of care and the optimal ANP role fit within the models of care, as conditions that will allow the ANPs to meet the patients’ needs.

In addition to literature about advanced nursing roles [1, 27, 28], decision-makers also have expressed the need for more information about different models of care involving ANPs [29]. PEPPA-Plus specifies that Donabedian’s model, distinguishing between structure, process and outcome evaluation, is applicable for ANP roles [12, 15]. We identified a number of studies that have applied Donabedian’s model on ANP models of care, and they were mainly from Australia [30–32]. More knowledge is needed about the ANPs models of care and their evaluation in the Scandinavian and European context, but also in a setting where the role is new (in Australia the ANP roles have longer history than Norway). This study is aiming to fill these gaps, by offering a structure evaluation of the models of care that were developed for the introduction of the AGN role in primary healthcare in Norway.

(35, 36)*Role* is a description of a person or a position’s behaviours, characteristics, norms and values [33]. In our study, the AGN role implementation happens within the models of care that the Norwegian municipalities are developing around the AGN role [19]. It is the model of care that defines the AGN’s patients and collaborators, as well as patients’ trajectories after AGN encounters. The model of care defines the goals, but AGNs’ competence and skills are the tools to achieve them. The AGN role in Norway recently has been described in other studies [14, 34], so this article focuses on AGN role implementation through models of care.

(39)In this article, we study the first municipalities in Norway that introduced and implemented the AGN role within a number of different models of care [12]. We also use the distinction of assessment of the models of care into structure, process and outcome assessment, in line with the PEPPA-Plus framework [35, 36]. This article focuses on the assessment of the structures of the models of care, which are defined as the ‘practical, human, physical and environmental factors that influence how ANP roles are implemented’ [12]. The structure assessment of the municipal models of care that are implementing the AGN role includes studying the models’ characteristics, with an emphasis on the AGN’s role in each model and the models’ potential to correspond to the population’s needs [12, 17]. Structure assessment assumes that good healthcare requires proper settings and instrumentalities [36]. Therefore, we consider the model’s ability to adjust its structure, settings and instrumentalities, which also are part of the structure assessment. We also use the integration of the model of care as one of the aspects for successful implementation of each model [1, 18, 35].

The integration of a role is defined as the inclusion of a role in the provided routine care [1, 34]. Based on that definition and the relevant literature [11, 12], the integration of the model of care within the healthcare system can be defined as the inclusion of the model of care in the routine care provided by the healthcare system [21, 36].

## Methods

### Objective

Our objective was to provide a qualitative structure evaluation of models of care developed to implement the AGN role in primary care by identifying conditions that affected the implementation of the different models of care. Furthermore, we compared how the impact of the conditions varied across models and municipalities.

### Design

We designed an embedded multiple-case study, with each municipality as its primary unit of analysis [37]. Each municipality was a separate case that helped us understand the development of the models of care around the AGN role, and the special characteristics that affected these models’ development. Our *quintain*—i.e., the phenomenon to be studied [38]—was implementation of the AGN role, and the multiple cases allowed us to study the quintain in different contexts. Some municipalities have developed more than one model of care either to cover different needs or because the first model did not work as expected. We considered these to be embedded units of analysis within the existing cases that—because they belong to the same municipality—do not comprise different cases. This paper focuses only on the structure assessment of the implementation of the AGN role, which is only one of the dimensions that our quintain could elucidate.

### Setting of the study

The University of Oslo started in 2011 to offer a master’s programme in Advanced Geriatric Nursing. It was a four-year part-time study programme that included advanced theoretical and practical training related to direct and indirect care, teaching, supervision and coordination functions [34]. The master’s programme mainly targeted primary healthcare, which in Norway is the responsibility of the 422 municipalities. Within a nationally regulated framework, each municipality has the freedom to organise health services provided to its citizens in its own way. These services include GPs, home nursing, nursing homes and public health services [39]. In our study, the municipalities have had the main role in designing different models of care to implement the AGN role in their primary care systems.

### Sample/participants

The municipalities that employed the first students from the master’s programme in Advanced Geriatric Nursing at the University of Oslo who were enrolled in 2011 were invited to participate in a project that would develop new models of care for the graduates and follow up with the current research study. Five municipalities, which employed six AGNs from the 2011 class, volunteered to participate in the project. One municipality later employed one more AGN (Class of 2013) who also was included in our study. The participating municipalities represent the early adopters of the AGN role, since they were some of the first in Norway to employ AGNs. These municipalities were located in the southern part of Norway and do not represent the context of other parts of the country, but do vary in population, from 5000 to 122,000 citizens. Three were urban, one was semi-urban and one was rural (Table 1). We interviewed seven AGNs, 10 municipal leaders, three Municipal Medical Officers, two GPs and one physiotherapist. Across cases the sampling was purposeful, aiming to include participants that could provide rich information about each case. Within the cases, the sampling was a combination of maximum variation and snowballing, meaning that within each model of care we aimed to interview different stakeholders, and that participants were asked to identify other stakeholders that could provide us with relevant information. At least three stakeholders per model of care were included, although in municipalities with more than one model of care, the stakeholders were overlapping. Recruitment was constrained by the small number of stakeholders familiar with the models of care and AGN role, and the stakeholders’ limited capacity. It has been particularly challenging to recruit GPs, and the reason might be lack of time. Nevertheless, data saturation was achieved, especially regarding cross-cases analysis.

**Table 1:** Overview of characteristics and models of the municipalities in 2016

	Population (approx.)	Urban/rural	Municipal economy^a^	Educational level^b^	Household income after taxes^c^	Number of AGNs
**Municipality A**	122,000	Urban	Good	High	123%	2
**Municipality B**	22,000	Urban	Average	Average	106%	2
**Municipality C**	11,000	Urban	Average	Average	91%	1
**Municipality D**	6000	Semi-urban	Average	Below average	94%	1
**Municipality E**	5000	Rural	Below average	Below average	91%	1

^a^ Based on the net operating profit as percentage of gross revenues in comparison to the national average. ^b^ Based on the percentage of population over 16 year old with higher education in comparison to the national average. ^c^ % of median national income

### Data collection

We used interviews, documents and statistical data, including data from nine interviews with AGNs and with 16 other stakeholders in primary care. They were semi-structured face-to-face interviews with two different interview guides. The first interview guide was used for interviews with AGNs and covered the AGNs’ experiences with the new role, the AGNs preparation for the new role, the description of the role, the description of the model of care, and the nature of the collaboration with others. The interview guide has been published earlier [14]. The second type was used for interviews with stakeholders and additional interviews with AGNs when needed and can be found as Additional File 1. The themes of the latter interview guide included the description of the care for older people in the municipality provided by the interviewed stakeholder, the role of the AGN in that municipality, the description of the model of care and who participated in its design, positive and negative consequences of the AGN role and the service for the patients and the municipality. The guide also suggested asking stakeholders about their thoughts for the future of the AGN role, general comments on the care for older adults in their municipality and experiences or thoughts regarding the AGN role in addressing older patients’ needs. Interviews lasted 45 to 60 min each, and most were conducted at the participants’ workplaces. We also collected documents related to the AGN role or the model of care, such as role descriptions, PowerPoint presentations created by AGNs and other stakeholders, information and news published on websites and municipal organisational plans and reports. Statistical data were retrieved from Statistics Norway and the Norwegian Directorate of Health. These data were used to achieve a better understanding of each case while creating the case reports and the Analyst’s notes. Table 1 is also based on some of the statistical data.

### Data analysis

The audio recordings of the interviews were transcribed verbatim, and together with the collected documents and the statistical data mentioned in the previous section, they were organised per case and subsequently per AGN. For part of the analysis, we used NVivo 11 (QSR International Pty Ltd., released in 2015). While collecting the various data, this paper’s first author started writing a case report for each municipality in the study. Each case report was presented to the AGNs of the respective municipalities for corrections and comments. We constantly updated the case reports based on the AGNs’ comments and as new data were collected.

The overall project covered more objectives than those presented in this paper. Following Stake’s approach [38], we formulated themes for the multiple-case study. The themes relevant to the objectives of this paper were: Actors involvement in each model during design and implementation, role clarity and consequences, adaptability of the model, and factors for successful implementation of the model (structure factors, populations size etc.). The first author used these themes to write the Analyst’s Notes from each case report, simultaneously rating each case’s prominence and utility concerning each of the themes as low, middle, or high. The ratings then were combined to create an overview of each case’s expected utility for each theme. The Analyst’s Notes also contained the findings from each of the cases that also were rated individually based on their importance for understanding the quintain through a particular theme.

The findings from the Analyst’s notes were studied together according to the existing themes, and based on them, we constructed some tentative assertions. Then tentative assertions were combined to construct the final assertions. At the end, we grouped and prioritised the assertions to be reported [38]. *Assertion* is a term used to describe a researcher’s proposed generalisation from vigorous interpretations of data from the multiple cases [38]. These generalisations refer mostly to the case study’s context (petite generalisations), but occasionally can refer to a wider context (grand generalisations) [40]. This publication presents the assertions that are related to the structure assessment [36].

We also used two of the three levels of social aggregation––the meso-level to refer to findings related to the municipal level, and the micro-level to refer to the model-of-care level. The macro-level would refer to nationwide findings and would be beyond the scope of this study. This approach is common in social sciences [41] and has been used in health policy research [42]. Finally, for the interpretation of our results we found the use of PEPPA and PEPPA Plus frameworks [11, 12] very relevant. As several assertions were emerging from our data, we found similarities to the issues discussed by those frameworks.

### Validity and reliability/rigour

We combined qualitative and quantitative data, and qualitative data from different sources to triangulate facts and findings and to improve the study’s construct validity [37, 38]. For example, the adaptation of a model of care in one case was both presented in the interviews with stakeholders, but also documented in detail in a report published by the municipality. We presented the case reports to the AGNs and asked for comments as a form of member checking [43]. Peer debriefing with a senior professor (last author) and a PhD candidate with good knowledge of AGNs’ education and role in Norway also was used to strengthen the study’s credibility and enhance the analysis of the data [43].

## Results

The objective of our study was to provide a structure evaluation of models of care developed for AGNs in primary care. The five municipalities included in our study developed different models of care around the AGN role (Table 2). One model was the Professional Development Nurse role, combined with direct patient care, in which the AGNs worked 50% clinically as AGNs and 50% with internal training and supervision. Another model was the Nursing Policlinic, in which the AGN leads a primary care policlinic for specific chronic diseases. The Response Team model of care supported home nurses with extra resources in complicated patient cases. The Virtual Ward model of care was a team, led by an AGN, whose objective was to make the patient’s transition from the hospital to home care services efficient and seamless. The Quality Coordinator model of care combined the AGN’s clinical function with extended responsibility for quality auditing and improvements in municipal health services. Finally, a model of care existed whose main focus was on the AGN’s direct patient care function.

**Table 2:** Description of models of care

Model of care	Municipality	Target population	Direct patient care	Other functions	Team members	Physical characteristics	Environmental characteristics
50% Professional Development Nurse 50% Direct patient care	Municipality A	Elderly patients who are in acute deterioration and new patients, including those coming from other healthcare levels (hospitals, nursing homes, etc.).	Clinical evaluation of target population, coordination and planning of offered services.	Internal training of the staff, a resource person for the unit and responsible for the district’s dementia care team	1 AGN	Colocation with district home nursing service. Delivery of direct patient care at patient’s home or nursing home.	Report to the district leader, hierarchically over staff nurses.
Nursing Policlinic	Municipality B	Patients with type II diabetes, chronic obstructive pulmonary disease (COPD), urine incontinence and wound management needs.	Users initiating contact were offered guidance about the available services. Routine control of type II diabetes and COPD patients. Assistance with wound management. Advice and guidance for incontinence. Two different service ‘packages’ for diabetes and COPD after referral by GP.	Regular internal training and supervision and informal training of colleagues.	1 AGN	Located at the local health centre (*Helsehus*). Users visit the policlinic.	Nursing Policlinic belonged to the municipality’s Home Services Unit. Home nursing work groups belonged to the same unit.
Response Team	Municipality B	Patients with dementia or other cognitive problems, weight loss, low physical activity and increased risk of falls.	Mapping and evaluation of target population.	Regular internal training activities. Supervision and informal training of colleagues.	2 AGNs 3–4 RNs 1 Physiotherapist	Colocation with home nursing services. Direct patient care through home visits	Response Team belonged to Home Services Unit and was a parallel structure to the four home nursing work groups. The Response Team was an extra resource for these work groups.
Virtual Ward	Municipality C	Patients over 65 years old with a hospitalisation that needs to be followed up by home services. Three diagnoses affecting function, with capacity to consent. No mental or addiction diagnoses.	Home visit by AGN after the discharge. AGN collects medical history and a medication list. AGN uses systematic clinical examination, and when relevant, blood, urine and bacterial culture tests. Follow-up visit by AGN up to 14 days later if deemed necessary. Physiotherapist also visits and evaluates patient. Creation of care plan by AGN, home nurse and physiotherapist, which is forwarded to GP.	The medical advisor held relevant lectures for the Home Nursing personnel.	1 AGN 1 Physiotherapist 1 Medical advisor Later adaptation included a GP	Colocation with home nursing services. Direct patient care through home visits.	Report to the manager of home based services. Virtual Ward was a parallel structure to 2 Home Nursing groups.
Quality Coordinator	Municipality D	Elderly patients with multiple diagnoses and multiple medications.	Systematic examination, systematic evaluation tools and discussion with patients and informal caregivers. Prioritisation of patient’s problems and creation of care plan. Examples of measures are fluid-intake monitoring, blood pressure monitoring or referral of patient to Emergency Department or hospital.	Quality surveillance and improvement, the internal training and supervision of personnel and the handling of deviations (*tilsynssaker*).	1 AGN	Colocation with home nursing services and nursing home. Direct patient care through home visits or nursing home visits.	The AGN is reporting to the manager of the Department for Service Allocation, Development and Innovation. Home nursing and nursing home are different departments at the same level.
Only direct patient care	Municipality E	Undiagnosed patients or patients with complications living at home or at the nursing home	Systematic physical and psychosocial evaluation of the patient, documentation of findings, creation of care plan.	No other formal functions.	1 AGN	First colocation with municipality’s administrative services. Eventually, colocation with home nursing services and GP’s office. Direct patient care through home visits or nursing home visits.	The AGN is reporting to the chief municipal officer for Healthcare Services. The Unit for Home Nursing and the two nursing homes also belong to Healthcare Services.

Our main findings that emerged as final assertions from the multiple case study are presented from two levels––a meso-level, which refers to findings related to the municipality level, and a micro-level, which refers to findings within the models of care. At the meso-level, we present both common and different structure characteristics of the models of care, stakeholders’ involvement in the design of the models of care, evaluations of the models, clarity about the models of care and their goals at the organisational level, and major adaptations of the models over time. At the micro-level, we present the collaborations within the implemented models of care, the AGNs’ role clarity and adjustments within the existing models of care. These findings address the study’s objective by identifying the most essential structure-related conditions that seem to affect implementation of the various models.

### Meso-level

#### Structure characteristics at the municipality level

In all the cases, the municipalities created models of care in collaboration with the AGNs. The process was facilitated by a network of leaders of the AGNs and coordinated by the University of Oslo. In this network, municipal leaders and academic personnel were sharing and discussing ideas and experiences, and in some cases, even concrete job descriptions for the AGNs and suggested models of care. A common characteristic of most of the models of care was the placement of the AGN at a higher hierarchical level than the staff nurses at the nursing homes and in home care services, but without management responsibilities. This meant that they could dedicate the time that each patient needed and did not need time restrictions placed on the regular staff. Also, some of them had the independence to select patients on their own. The AGNs also were included in meetings at the leadership level. On the other hand, AGN positions were vulnerable to organisational changes (e.g., a reorganisation), and in most cases, they were more expensive than staff nurses. One of the leaders addressed this view in the following way:

‘What I was very concerned about because of the hectic routine both at the nursing home and the home nursing, was that [the AGN] wouldn’t be under one of these departments, but independent…[she] had [an] office with the home nurses… but they would not decide her daily routine… I have strong faith in such [a] structure, so the person does not get consumed by the [daily] operational routine…’ [Leader, Municipality E].

#### Stakeholders’ involvement in the design of the models of care

Most of the municipalities in this study intended to include a wide variety of stakeholders in the design of their models, and this was successful to a certain extent. However, our data suggest that the resulting involvement in some cases might not have been substantial enough, i.e., some stakeholders were not involved, were only partially involved or were involved, but did not feel like their voices were heard.

The managers of the different municipal services were important stakeholders who were involved in the design of the models of care. The municipalities’ top-level management and the unit (low-level) management were, in most cases, actively involved, or at least well-informed, about the design of the new models of care. This seemed to play an important role in the successful implementation of the models of care, as well as in providing the support that each AGN needed. As one AGN recalls:

‘I am a bit unsure what the leadership of the municipality actually expected when I came back. I almost cannot give a certain answer, but in connection with the fact that we started with the model of care, it was anchored high in the leadership, all the way up to the mayor and the Health and Social Committee’ [AGN, Municipality C].

The GPs’ involvement did not seem to be optimal in the design phase of the models of care. The reasons varied, and in some cases, despite being invited to participate, they did not. The project report on the Virtual Ward describes the problem and its implications:

‘The project team is self-critical for its relationship to the GPs. If they [had] been better involved and informed in the first place, maybe they would be more engaged participants in the project. For example, we wished to have a GP [on] the project team, but we didn’t succeed. If we had been prepared to provide financial compensation for lost income to the doctor in connection to the meetings of the project group, the recruitment might have been easier’ [Project report, Municipality C].

In the largest municipality in our study, managers’ involvement at different levels seemed to leave a gap in middle-level management, as these leaders were not involved in the design of the models. According to the AGNs, better knowledge about the role across management levels could have improved the level of integration of the new models of care.

In one municipality, the GPs seemed to be concerned about the introduction of AGNs, creating fragmentation in primary care. The interview with a GP from that municipality revealed that the reason for these concerns did not involve the AGN’s role, but the way that the AGNs were used in that municipality, which, in our study, we interpreted as the model of care. This view was not shared by other stakeholders or the AGNs in the municipality, and it might be related to the pre-existing close collaboration between the GPs and the home nursing teams. Unlike other municipalities, each GP was collaborating with one home nursing team for his or her patients to enhance collaborations. Interestingly, that municipality had the most systematic approach among our cases, including GPs in the development of the model of care. The GPs were included in the design phase of the model of care, but they were not positive about it. The GPs felt that their concerns were not heard:

‘[In the design phase of the nurse policlinic], there were many GPs in those groups discussing the COPD (Chronic obstructive pulmonary disorder) policlinic, dementia policlinic, chronic-wound policlinic––several policlinics, and I was then also part of it. But the GPs’ clear recommendation was that there should not have been so many policlinics because then we, the GPs, will lose the oversight […]’ [GP, Municipality B].

When the same municipality decided to use the AGNs in another model––the Response Team––the GPs felt like they were not invited to express their opinions. One of the GPs described their involvement in the following way:

‘We, the GPs, have not been involved in how the AGNs will be used in our municipality [regarding the Response Team]. We have not received a question about it at any time. When it comes to the organisation of the Response Team, I am a bit unsure whether it is suitable because it contributes to the fragmentation of primary healthcare’ [GP, Municipality B].

The other nurses employed by each municipality were also important stakeholders because of their role in the provision of primary healthcare. They were Registered Nurses with and without specialisation training to work in home nursing services and in nursing homes. AGNs also supervised or collaborated with nursing assistants. Neither nurses nor nursing assistants had much involvement in the design of the models of care. This was also the case with physiotherapists and occupational therapists, despite their frequent collaborations with the AGNs. Other professionals’ limited involvement is apparent in the following quote:

‘[In the development of the model of care], it was me who was involved all the time, together with my leader, the unit leader. We did it together and in collaboration with the university because we were part of a project. My role developed as time passed, since I have gotten more and more tasks, so it is difficult to point out when the role was developed. […] And there have been others, department leaders, but mostly me and my leader, that in a way have developed, discussed the role a lot and added new responsibilities. But it has been mostly me creating my own path. As if it is only me that knows what can be my responsibility’ [AGN, Municipality D].

Our data also show that the patients, as potential users of the municipal models, had little involvement in the design of the models, as only one of the studied models included patient representatives in the design of the models.

#### Clarity of the models of care and their goals at the municipal level

Among the municipalities in our study, only the largest of them developed a job description for the AGN in the model of care, and it was officially approved by the municipality. Nevertheless, one of the AGNs of that municipality experienced this document as not being helpful and desired an even more concrete and clear role:

‘The municipalities that are lucky to have one or more nurses with our qualifications [i.e., a clinical master] I believe should have expected more from us, and achieve a better collaboration by being more specific. Instead, it has been quick solutions. […The job description] was very vague, and it has been like this since I started; it is very vague. I am supposed to work 50% clinically toward the patients and 50% in professional development around the needs of these patients. The reality is that I am working a lot with professional development generally in the district here. I do a lot of internal training, and I have the responsibility for the annual programme for the professional subject that will be in focus each month, things that I believe I shouldn’t have been spending my time on. I should rather do more clinical evaluations and other similar things––these type[s] of things I ought to be used for’ [AGN, Municipality A].

The existence of concrete descriptions of the model of care varied among the other municipalities. In one municipality, the model of care and its adaptations very clearly were defined in reports that were published approximately one and 2 years after the model’s implementation. In another case, the model of care and the AGN functions were documented when the AGN was to take a leave, so the municipality could plan for different substitutes for the various AGN tasks. We also have had access to several PowerPoint presentations that described the different models and were used to present the models of care to colleagues inside and outside the municipalities:

‘In addition, we created a PowerPoint [presentation]; it sounds a bit funny, but we created a PowerPoint in which we included more than just the job description. Because we had to promote this to the [home nursing] service, we had to make it visible internally in the organisation’ [Leader, Municipality A].

The models of care did not seem to be very clear to several primary healthcare actors inside and outside the municipality. For example, the middle-level management of the largest municipality in our study had limited knowledge about AGNs and the models of care. In many of our cases, the other nurses in the municipalities, the GPs and other health professionals did not distinguish between the models of care and the AGN role, and did not know enough about any of the two:

‘It was a lot like “What are you really doing?” It was, of course, in the beginning “What is your thing?” It was like nobody understood, not my nursing colleagues, not the doctors’ [AGN, Municipality C].

‘If you had asked one of my clinical colleagues, [maybe they would think that role could have been more visible]. Maybe they don’t know enough about it…I think that information was spread to the home nursing personnel, but I am [a] bit unsure how much information has reached other units that have other professionals. If you would ask one of the other physiotherapists, I am not sure that he could say [as] much about the AGN role as I can’ [Leader/physiotherapist, Municipality D].

Except for the one municipality that published evaluation reports, we observed vagueness when it came to expected goals and outcomes from the models of care. Despite the fact that several leaders mentioned the existence of goals, they were often very general and not necessarily related to the AGN models of care:

‘No, I think that [we do not have concrete goals] related to the AGNs. We do have focus areas for care in our municipality. It is dementia, it is palliation and rehabilitation––reablement. Those are the main focus areas. When the municipality is subsidising education [for nurses], it is mainly for education in these focus areas’ [Leader, Municipality A].

#### Evaluation of the models of care

The AGNs had a strong feeling about the positive impact of their work. The leaders of the AGN also were positive about the AGNs’ work, and they were enthusiastic regarding their impact on their municipalities. Despite this positive attitude and the enthusiasm, in most of cases, neither the AGNs nor their leaders could identify quantifiable improvements related to the models of care and AGN functions. They had a general impression that they made a positive impact on several levels, but often without documentation to support them:

‘We don’t have numbers to say as it is. I cannot claim that it has been a reduction of so many percent. But we saw that the AGN role was important in some cases [so] that the users did not return to the hospital. Instead, they started treatment at home’ [Leader, Municipality A].

‘No, [we do not have any specific numbers]. The AGN was supposed to write a log, and she did it…and she was supposed to write a report, but then she started as a PhD candidate and is very busy. She has the overview, but it is not ready…When I had to propose the budget for this position, I was not able to find the numbers. But in the aftermath, I feel that I managed to explain why this is such an important competence for the municipality. So, it is not that I had to have the numbers’ [Leader, Municipality E].

As several models were adapted over time, we can assume that a form of evaluation has driven these adaptations. Nevertheless, in only one of the models of care included in this study could we find evidence of systematic evaluations and reporting by the municipality. In the municipality that implemented the Virtual Ward, the AGN, in collaboration with a researcher, published a report on the experiences and the results from the first year of the model implementation [44]. Based on these results, the model was expanded to a neighbouring municipality, and a new report was published with results from the expansion [45]. Two other municipalities seemed to have started the preparation for systematic evaluations, but they seemed to have stopped (or remained unpublished and unarchived) because those responsible for them moved to different jobs. The existence of the reports on the Virtual Ward might indicate a more conscious inquiry of that municipality to understand how the role and the model of care can be used, compared with the other municipalities:

‘It is worth mentioning that [Municipality C] as [host of the] Development Centre for Nursing Homes and Home Nursing [in our greater area] from 2017 wants to be a driving force for the further development of the Virtual Ward. The intention is that the Virtual Ward will continue to focus [on] increasing the professional competence of the health services of the municipality, build good collaboration platforms internally, but also toward hospitals, health centres and GPs’ [Report, Municipality C].

#### Adaptation of the models

The models of care in almost all the cases were dynamic and able to adjust to the patients and municipality’s needs, and to adapt to changing needs. The first factor that we identified was the degree of adoption and utilisation of the model. Usually, this was not based on quantifiable results, but on the impressions of the AGNs and their leaders (as pointed out above). The nurse-led policlinic seemed to have received only a low number of users, so the municipality quickly decided to change to another model. In the case of the biggest municipality in our study, while the model of one of the AGNs received a satisfying stream of patients and did not get adapted over time, the model of another AGN was adapted as a response to inadequate utilisation of the AGN role. One reason might be that an important element of the common model of care in the two instances (the dementia team function) was, in the one instance, already established before the introduction of the AGN role. That element seems to have functioned as a good source of patients there. In the other instance, the same element did not function optimally, and as a consequence, the AGN only received a small number of patients.

Another factor that can lead to adaptation or changes in models of care is the internal changes that may happen at the municipal level. For example, in the largest municipality in our study, there has been a reorganisation of the districts covered by each home nursing team, also causing the need for adaptation in at least one of the AGN models of care. Toward the end of data collection, the second-largest municipality in our study merged with a neighbouring municipality, forming a new municipality. Consequently, the Response Team’s population catchment and geographical coverage increased. Similarly, the models of care are sensitive to changes in external factors. For example, there have been changes to the funding scheme for physiotherapy care within primary healthcare. For the Virtual Ward, this caused great concern for the AGN, as it would affect vulnerable Virtual Ward patients’ ability to receive care from a physiotherapist.

Some of the models did change, even if the level of adoption was high. In these cases, it seems that the goal was optimisation of the models to improve their performance. For example, the Virtual Ward expanded into a neighbouring municipality because the AGN had the capacity to cover larger populations. Due to the neighbouring municipality’s limited utilisation of the Virtual Ward, mainly due to implementation issues, the expansion was revoked after the first year. Some implementation issues included the different organisational structures, the different services provided by the two municipalities and the lack of available resources to support the Virtual Ward in the new municipality due to inadequate access to necessary personnel.

### Micro-level

#### Collaboration within the implemented models of care

The AGNs found that through their education, they had become better at collaborating with other professionals. Their skills in collaboration were utilised in the models of care that often would require a good understanding of what other professionals or other services can offer, and how to communicate effectively with them. As one AGN explains:

‘I experience it very clearly that I have another way of meeting with other professionals. I am being heard in a completely different way by other professionals––now, I am answering to what I am supposed to. So, yes, it is the clinical; it is the knowledge, the competence, from this seat, I can see that I can contribute to my working environment by being––how should I say it––a leader in my profession, a driving force professionally and that [I] contribute there in a way that is both clinical and professional. I am asking critical questions about what we do. I am trying to come up with questions, not only that I am asking them, but also that we move a step further. What are we doing with these things? The same applies to the patient and the relatives; you can take the next step and get a grip on the issue. You get different approaches to things’ [AGN, Municipality D].

The collaboration between AGNs and GPs often took time to establish, as the GPs needed time to trust the new type of competence that AGNs represented. GPs’ involvement in the implemented models of care in our study usually started on a limited level. The AGNs were contacting the GPs by phone, by electronic messages or in person, and they were discussing with them patient cases and would send reports to them. In this way, the GPs gained increasing understanding in the AGN’s role and its potential:

‘I see that the [AGN] role is very important because she is a contact person who can interact with these GPs. I know that there is some way to go until those GP offices get to know [the AGN] because they were sceptical initially. But it improved when they understood that the [AGN] was not going after their job’ [Leader, Municipality A].

The GPs included in the study clearly appreciated the AGNs’ knowledge and skills, and they saw the need for better nursing competence in the municipalities:

‘I experience that she has a more systematic way of thinking, and it is easier to get into a dialogue about principles and systems. She has a more academic way of thinking. So, my experience is that she absolutely [is] a resource’ [GP, Municipality D].

‘[The two AGNs] are very skilled nurses that I trust very much. They have high competence in their nursing field, and they can support when I need it, and this––I believe––is good. I believe that it is good that everybody that we collaborate with have as [much] competence as possible, so this is good’ [GP, Municipality B].

In some cases, the GP-AGN relationship reached a high level of mutual trust, and GPs based their decisions on AGNs’ reports. When this level of trust was achieved, AGNs reported how the collaborative atmosphere improved:

‘Yes, I believe it has become better, but it takes time because…doctors are not so happy to be told what to do by nurses. But they do listen, some time passes, they think about it, and maybe you get an electronic message that says, ‘Yes, let’s do it, OK’ [...]. It takes time, and also, in a way, you should know where the limit is because it is, indeed, the GP that has the medical responsibility for the patient, but I also know the patient quite well, I see them more often than they do, and the patient [says] more to me [than] to their GP. […] But it takes [a] long time to be trusted; it also costs a bit [emotionally]. […] The GPs, in a way, have understood quite fast what I can do’ [AGN, Municipality D].

The inclusion of other nurses and nursing assistants on the team for the model of care varied, but they were, in all cases, important collaborating partners with the AGNs and their models of care. The collaboration also was close and solid with the physiotherapists and occupational therapists working in the municipality, and in some cases, they were considered members of the team implementing the new models of care:

‘The AGN has been a driving force for interdisciplinary collaboration. She sees the importance of interdisciplinary collaboration, [..] and interdisciplinary collaboration is not as simple as someone would believe. And someone can see that we have different glasses on when someone meets the patient; [we] have different strengths, and together we do a good job. It has not been taken for granted here. […] The other nurses in nursing homes or home nursing, they are so busy, they have so much to do, that it becomes difficult to achieve a good enough interdisciplinary collaboration, mainly because of busy times. I experienced that the AGN has the extra time to do these evaluations in a good way’ [Physiotherapist, Municipality E].

Finally, the AGNs were emphasising that the care offered through the models of care was user-centred and that they were collaborating with the patients to empower them to participate in the decisions regarding their own health:

‘The change [in] direction that we are working with [at the national level], from asking [the patient] ‘What’s wrong with you?’, thinking of diagnoses and sickness…to ask the patient instead, ‘What is important for you?’ This change of direction […] has not been so widespread in our municipality, but an AGN is very concerned about this’ [AGN, Municipality E].

‘I was asked what I want. Not only what others had said that I should get’. [Patient quote from project report, Municipality C]

‘I was allowed to say what I wanted. I could decide myself what I needed help with’ [Patient quote from project report, Municipality C].

#### AGNs’ role clarity

In some cases, the models seemed to be unclear. The AGNs themselves had difficulty defining their role in the model, and in almost all the cases, there were collaborators who had difficulty understanding the model of care and the AGN’s role in it. This was indicated by some GPs’ limited understanding of the models’ clinical function, along with some of their leaders, who, during interviews, only provided a vague description of the model of care. Their uncertainty could be related to the AGN’s position in the municipality and the models’ dynamic nature. Nevertheless, all the AGNs expressed certainty as to what their direct care duties were, as well as confidence about their ability to use their knowledge and skills as AGNs:

‘An AGN is someone with expanded clinical competence, and a core part of it is the patient evaluation competence, and the AGN focuses on geriatrics, the older people. Yes, I believe that I start becoming good in evaluating older people. I have the competence for that, I am sure about this. I have––how should I say it––grown into using it through my studies. I am also reading a lot […] So, patient-evaluation competence and evidence-based acting––I believe that it makes you feel safer when you meet others; you become a stronger interdisciplinary profession despite the fact that you have been in your own profession and you have specialised in it. […] The clinical [function], the competence, the supervision, the approach, it is a generic competence that I believe characterises a typical AGN. You are more independent, and at the same time, you work in a more interdisciplinary way. You are going into situations and you plan, think, evaluate…There is an independence in this also’ [AGN, Municipality E].

#### Adjustments within existing models of care

In contrast to the models’ adaptation at the meso-level, there were also adjustments within the models of care that aimed to optimise their function. The Virtual Ward eliminated the lower-age-limit requirement, reduced the number of active diagnoses required for referral from three to one and included a GP on the team of the model, thereby increasing access to the AGN for a larger group of patients. The rationale for these adjustments is explained here:

‘This means that in practice, “we made the way by walking”. Along the way, we experienced that not everything that sounds good in theory is the same and [easily] realised in practice. To work with people is, in essence, unpredictable. Nevertheless, it is important to create a model and a procedure to make sure that services are prudent and of good quality’ [Project report, Municipality C].

In the smallest municipalities of our study, the relocation of AGNs’ offices from the municipal administration headquarters to the building that housed home nursing services improved collaborations with home nursing. The adjustment of the AGN’s working hours to include the coordination/handover meeting for home nurses’ morning shifts also seemed to improve the number of referrals of patients to the AGN.

In the initial model of the largest municipality in our study, home nurses and GPs were expecting to refer patients to the AGNs. Due to the non-optimal implementation of the model in one of the districts, the referral routine for that district was adapted, and the AGN started choosing patients herself from the list of new patients in the district and those who came back after hospitalisation. This seemed to increase the number of patients referred to the AGN and is in agreement with other observations that we made that favour the self-selection of patients over referrals by others, at least in the early implementation period.

## Discussion

It seems that several structures necessary for the successful implementation of the AGN role through the models of care in our study are, indeed, in place, but simultaneously, a clear potential exists for improvement. Stakeholder involvement could have been broader, the models of care and the AGNs’ roles in the models could have been more clear, and each model’s goals could have been more specific. Another common characteristic has been the smaller or bigger adaptations of the models of care, suggesting difficulties in integrating the models of care and possibly contributing to better integration of the models of care. Finally, we did not find evidence of a systematic evaluation of the outcomes of the models of care in most of the municipalities.

Not all the stakeholders were involved in the design and implementation of the models of care to the extent recommended in the literature [11]. The PEPPA framework argues for the inclusion of all potential stakeholders, from a methodological perspective, and they associate problems related to role clarity, boundaries, role acceptance and potential barriers to the process of stakeholders’ involvement. Our empirical observations at the meso-level support these arguments. Even though medical doctors in different functions were involved in the design of many of the models, local GPs’ involvement during the development phase was limited, and sometimes they were not satisfied with the model of care in which the role was implemented. Strong nursing orientation of ANP roles has been associated with optimal outcomes, but this might be challenging traditional medical models [11]. Reactions to new roles and new models of care are expected; therefore, it is important to follow a structured and inclusive process.

Limited clarity in the models of care, in the AGNs’ role in them and in the models’ goals was prominent in our findings. The PEPPA framework suggests that all the stakeholders should define the ANP’s role in relation to the other care providers and define the model’s related functions for the ANP [11]. Our results show that these issues were not clarified from the beginning in many of the models, contributing to role ambiguity and role conflict at the meso-level [46]. In some cases, the models of care and the AGN’s role in them were documented at some point. It might have been beneficial to have had earlier adoption and integration of the models of care to define them and make them available to all involved actors from the beginning.

The employer’s understanding of the role seems to have an important direct influence on team dynamics and an indirect influence on the clinical dimension [15], and a supportive attitude by the administration is a prerequisite for successful implementation of advanced nursing roles [16, 47]. In our study, we met enthusiastic leaders with high expectations for AGNs and their models, but their understanding of the AGN role and the models varied across municipalities and across management levels. As another study suggested, the eagerness to introduce new models of care might lead to people being overly optimistic about the anticipated future savings, as well as the time frame for implementation of these new models [48]. A realistic approach can be more beneficial, as it is more likely to inform a successful implementation strategy with clear, measurable and achievable goals.

Role clarity also is related to the professional, educational, organisational and healthcare system policies that are in place [12]. Our data show that the lack of professional and healthcare system policies regarding the AGNs in Norway possibly is contributing to the challenge of integrating and establishing this new role [14]. The AGN roles also are affected by budget pressures that municipalities in Norway face, confirming older US studies’ observations [47]. The PEPPA-Plus model also mentioned funding as an important aspect in the implementation of ANP roles [12].

The existence of a job description has been found previously to exert a negative influence on the clinical dimension of the Clinical Nurse Specialist’s practice, but the authors did not have data to explain the mechanism [15]. Only one municipality in our study had a job description for the AGNs working there, and we do not think that its existence there positively or negatively influenced the AGN practice’s clinical dimension. Another study emphasised the importance of role clarity as an organisational process and a professional competency of the nurse practitioner, but did not connect the clarity of roles with the existence of a job-description document [19]. The same study also argues that the roles are dynamic and should be redefined over time.

Our study found that municipalities put a limited focus on the structured evaluation of activities and outcomes of the models of care, in contrast to the emphasis that existing literature put on it [11, 47]. There are several examples of the impact of evaluation on maximising ANP roles’ potential and long-term sustainability [49], and the AGN’s role being only in its first years in Norway could benefit from systematic documentation of the outcomes of the role and of the models of care. The current study was part of a larger research project that aimed to evaluate the AGN role and the respective models of care. However, we suggest that future evaluations of the impact from the models of care also should be the respective municipalities’ responsibility.

We also explored the dynamic nature of the models of care and the factors that led to their adaptation. Despite its apparent necessity, the adaptation was not structured as suggested by the PEPPA framework [11]. For example, in most of our cases, there were no quantifiable goals or expected outcomes to use for the evaluation that would then inform the adaptation. Furthermore, in the one case in which the evaluation happened in a structured way, the evaluation informed the adaptation of the model of care, but without the same success as the original model. It seems that the first model’s evaluation phase was followed directly by the second model’s implementation phase, thereby skipping the development phase, as the PEPPA framework described. This leap might have led, as a consequence, to limited adoption of the new model.

Even if the adaptation of the models did not happen as described in extant literature, the fact that the models of care were able to adapt quickly might have had positive consequences. The capacity to change is considered a good characteristic for an organisation [50, 51], but we do not know whether it was a pre-existing characteristic of the municipalities, or if it was the AGNs or the stakeholders around them who acted as catalysts for the capacity to change. We observed that most of the AGNs were eager to adapt, and that the process of adaptation was easy to initiate. This was a characteristic of many of the cases, regardless of the municipality’s size.

### Implications

Our findings have several implications for future research. Wider stakeholder involvement, better role clarification at national level and clarification of the models of care at local level are some of the possible improvements that can contribute in overcoming the challenges we identified in this study. In addition, the ANP role and the ANP models of care should be regularly evaluated both at the national and the local level. Good examples for national level evaluation are nationwide workforce studies, as improving the availability and quality of about ANPs will lead to better monitoring of the ANP workforce [1]. These studies should also collect information about ANP models of care, in order to be able to better understand and document their impact on patient care. Our results also show the relevance of the PEPPA and PEPPA-Plus frameworks in the implementation of ANP roles, but more importantly, confirm the applicability of those frameworks to the evaluation of ANP models of care [11, 12]. The same applies to the Donabedian’s model [36], that although explicitly mentioned in the PEPPA-Plus framework, its use in this context was not common. We also found very useful the use of the levels of social aggregation [42], and they could be a valuable enhancement of the PEPPA-Plus framework. For the local level, the PEPPA-Plus framework can offer useful guidance. Case study methods such as the one used in our paper, can also be relevant for in depth understanding the implemented models of care. Finally, future research should be clearer in distinguishing between the ANP roles and the models of care, although there is certainly an overlap between them.

### Strengths and limitations

This paper is one of the first that study the models of care involving AGNs in Norway. Since it is a structure evaluation, it can provide an early insight on the potential of those models of care to improve primary health care for older adults, even before the outcomes of these models become visible or possible to detect. Therefore, it provides useful knowledge that can be used for the further development of the models of care, but also of the AGN role. As a multiple case study, it also provides a detailed and in-depth description and analysis of the implemented models of care, that can have national, Scandinavian and international relevance. We have distinguished between the AGN role and models of care that implement the AGN role, and we have focused on the models of care. This allowed us to study in detail and compare the different models of care, but also the different approaches that different municipalities have used to develop and implement the AGN models of care. Other strengths of our study are the triangulation of the data of different type and from different sources, the inclusion of stakeholders from different disciplines, and member checking with the AGNs.

This case study, despite including multiple cases from different municipalities, did not cover all the models of all the municipalities that implemented the AGN role in Norway. The selection of the sample is not representative of the Norwegian municipalities, and not all early adopters of the AGN role agreed to participate in the study. The fact that these municipalities are early adopters of a new nursing role might be characteristic of their innovation capacity and other positive traits that might be confounding our observations. Patients, as receivers of care, were not included as the AGNs suggested that it would be challenging to interview most of them because of their health condition, and cognitive status. Although an important viewpoint for the overall evaluation of models of care, this paper is presenting a structure evaluation. As such, we study the factors, settings, and instrumentalities related to the implementation of the models of care and the contribution of the patients to that might have been limited. In addition, due to their dynamic nature, the models have changed several times and are still developing. We have tried to document as many changes as possible, but we might have missed developments, between observations and interviews, that were not documented or communicated by the informants.

## Conclusions

The AGN role has been implemented in the diverse settings of five municipalities in Norway in ways that can lead to positive impacts for patients and the municipalities. The level of integration of the models of care varies across the cases, and most of the municipalities used the AGNs in ways that reflected their advanced knowledge and skills. There is certainly room for improvement when it comes to involving stakeholders in the design of the models of care, and particularly of patient representatives and GPs. This also will contribute to improving the clarity of roles that also was observed to be a challenge across the cases. Furthermore, AGNs, their leaders and the municipalities should work intensively to define clear and concrete goals (some should be quantifiable), as well as develop a comprehensive evaluation process that will capture the impact of the AGN role and of the implemented models of care. We evaluated the dynamic and adaptable nature of the models of care as a positive quality and a contribution to service innovation. However, adaptation should be driven by evidence to avoid premature rejection of some models.

## Supplementary information

**Additional file 1.** English translation of interview guide.

## Data Availability

Data generated or analysed during this study will be archived by the Norwegian Centre for Research Data (NSD, www.nsd.no) and will be available by NSD or the authors according to NSD’s sharing policy.

## Abbreviations

AGN: Advanced geriatric nurse
ANP: Advanced nursing practice
COPD: Chronic obstructive pulmonary disorder
GP: General practitioner
PEPPA: Participatory evidence-based patient-centred process for ANP role development, implementation and evaluation
PhD: Doctor of philosophy
US: United States of America
