# Supplementary material for: Structure evaluation of the implementation of geriatric models in primary care: a multiple-case study of models involving advanced geriatric nurses in five municipalities in Norway
Source: BMC Health Serv Res. 2020 Aug 14;20:749. doi: 10.1186/s12913-020-05566-y (PMC7427927; doi:10.1186/s12913-020-05566-y)
Supplement: Supplementary file 1 — Additional file 1. English translation of interview guide. [file 12913_2020_5566_MOESM1_ESM.rtf]

Additional file 1
English translation of interview guide
1.	Can you describe how the care of older adults is organised in your municipality?
2.	The role of the Advanced Geriatric Nurse (AGN) was recently introduced in your municipality. Which role does the AGN have in your municipality?
·	Is the AGN having new responsibilities/tasks or is taking responsibilities/tasks that someone else had before?
·	Which responsibilities are we talking about?
·	If the AGN has taken responsibilities/tasks from others, who had them before?
3.	Who did develop the role/model of care that the AGN is part of in your municipality?
4.	How does this model look like?
5.	How was this model developed?
6.	Have you observed or heard any positive or negative consequences/results of the AGN role/model of care for the patients of your municipality?
·	In addressing patients' needs?
·	In following-up of medical treatment?
·	In providing the necessary services?
·	In health promotion/disease prevention activities etc.
7.	Have you observed any positive or negative consequences/results of the AGN role/model of care for the municipality?
·	Economy?
·	Collaboration with others within the municipality?
·	Collaboration with the hospital?
8.	Which thoughts or plans does your municipality have about the future of the AGN role?
9.	Do you have any other comments about the care of older adults in your municipality?
10.	Do you have any other experiences or thoughts about the AGN role, related to addressing the older patients' need for care and follow-up?
Eventually other questions that can illuminate the model of care based on data that are collected or on data that are missing.
